# Editorial: Lipids in host microbe interaction

**DOI:** 10.3389/fcimb.2022.1002856

**Published:** 2022-08-23

**Authors:** Edith Porter, Juan-Carlos Saiz, Joseph T. Nickels

**Affiliations:** ^1^ Department of Biological Sciences, California State University Los Angeles, CA, United States; ^2^ ZOOVIR Dpto. Biotechnology, Instituto Nacional de Investigaciones Agrarias-Consejo Superior de Investigaciones Cientificas (INIA-CSIC), Madrid, Spain; ^3^ Institute of Metabolic Disorders, Genesis Biotechnology Group, Hamilton, NJ, United States

Lipids are increasingly recognized as important players in host microbe interaction. Host derived lipids comprise innate immune effector molecules with antimicrobial activity (Porter et al., 2015; Fischer, 2020), lipids with proinflammatory action, and lipids with anti-inflammatory pro-resolving function (Panigrahy et al., 2021), all of which could be used to inspire novel drug design to combat infectious diseases or promote normal microbiota. In some instances, microbes subvert host derived lipids to their own advantages and interfering with host lipid metabolism may offer gateways to novel approaches in controlling infectious diseases (Martín-Acebes et al., 2016; Martín-Acebes et al., 2019). On the other hand, lipids play also important roles in microbial physiology and a better understanding of their regulation and function in microbes can open distinct approaches to novel antimicrobial drug design (Gallo-Ebert et al., 2014). In the context of infection, the damage to the host is to a great deal founded in the inflammatory host response to the microbes. However, infection is not the only cause of inflammation. For example, the pathology of autoimmune diseases involves inflammation and can be linked to an imbalance of pro- and anti-inflammatory lipid action (Das, 2022). Therefore, manipulating lipids in non-infectious inflammatory diseases may also reveal novel applications for infectious diseases (Serhan, 2017). Considering that blood levels of metabolites reflect metabolite production and consumption in the periphery, it is not surprising that overall lipid profiles in blood are affected by infection and inflammation (Feingold and Grunfeld, 2022) and are considered as biomarkers and predictors of patient outcome.

The six articles selected for this Research Topic examine various aspects of the highlighted role of lipids in host microbe interaction. Mudgil summarizes the antimicrobial role of lipids in the ocular environment with special attention given to tears. The composition of the tear lipids, mainly contributed by the Meibomian gland, is presented and compared to lipids found in other body sites and secretions. The antimicrobial spectrum of the various tear lipid components organized by the various lipid classes is detailed and possible modes of action including synergism with antimicrobial peptides are discussed. This article gives an excellent comprehensive review of host derived lipids with direct antimicrobial activities.

Disrupting lipid synthesis in pathogens may offer novel approaches for overcoming the challenges posed by antimicrobial resistance. Zhou et al. set out to characterize substrate binding of the phosphatidylserine synthase Cho1, an enzyme linked to virulence and found in multiple yeast species, including *Candida albicans*. In this study, an alanine substitution mutagenesis approach was taken to map and characterize residues in Cho1 that affect binding of the two known substrates, cytidyldiphosphate-diacylglycerol (CDP-DAG) and serine. A critical residue was identified that was required for serine binding. *Candida albicans* strains expressing the corresponding Cho1 mutant allele displayed a slow growth phenotype. This now lays the framework for in vivo studies aimed at determining how altering serine binding affects virulence, while possibly spearheading novel drug discovery for new anti-fungal therapeutics.

As much as host lipids affect microbes, microbial derived lipids also affect the human host. Zhang et al. show in a rat model the effect of orally administered bacterial rhamnolipids obtained from *Pseudomonas aeruginosa* on lipid metabolism, immune response, and gut microbiota. The authors found that rhamnolipids reduced adipocytes at various body sites, reduced triglycerides, low density lipoprotein cholesterol, and non-esterified fatty acids in serum, while increasing high density lipoprotein cholesterol, thus causing an overall healthy lipid metabolic profile. The serum concentrations of the proinflammatory cytokines IL-1β, IL-6, and TNF-α were statistically reduced by the rhamnolipids. Finally, the authors also showed that the two rhamnolipids used significantly increased the α-diversity of the colonic microbiota and modulated the microbiota at the phylum and genus level in a distinct manner. This study reveals that microbial derived lipids play a role also in microbe – microbe interaction.

Alteration of host lipid metabolism may be also induced by viral infections. Jin et al. examined changes in the blood lipid profile in COVID-19 patients through two retrospective studies in the Chinese population in the search of identifying risk factors that are linked to poor outcome in COVID-19 patients. The authors found that elevated levels of total cholesterol and low-density lipoprotein cholesterol in both cohorts and triglyceride levels in one cohort were linked to poorer outcomes. However, the authors caution that organ dysfunction could contribute to the observed alterations in the lipid profile associated with poor outcome since markers of organ dysfunction positively correlated with lipid levels in contrast to the inflammation marker C-reactive protein, which negatively correlated with the lipid levels. This article proposes a model for the bidirectional interaction between host lipid metabolism and SARS-CoV2 and underlines the connection between host derived lipids and viral infection.

Finally, two studies examining the relationship between autoimmune disease and lipid levels in serum have been included in this Research Topic to put attention to potential shared pathways of lipid modulation in infectious diseases and non-infectious origin of inflammation. This could pollinate novel therapeutic approaches in each field. However, Wang et al. determined that there was no causal relationship between blood lipids, specifically high density lipoprotein cholesterol, low density lipoprotein cholesterol, triglycerides and total cholesterol, and the risk of systemic lupus erythematosus, an autoimmune disease. The authors used bidirectional two-sample Mendelian randomization and regression-based multivariate Mendelian randomization on a data set with single nucleotide polymorphisms from European individuals. In contrast, Andersen and Vance, based on serum concentrations, did find an association, though sex specific, between serum lipids and antinuclear antibodies (ANA), a hallmark of systemic lupus erythematosus and other autoimmune diseases, using an US study population. They found that a higher proportion of ANA+ women had high total cholesterol when compared to ANA+ men and that elevated serum triglyceride levels were less likely in ANA+ compared to ANA – individuals. Furthermore, women who were taking statins were less likely to be ANA+, which pushes forward the use of statins to reduce infectious disease mediated inflammation.

In summary, this Research Topic provides new insight into some of the many roles of lipids in host microbe interaction and unveils that perhaps host-microbe interaction is a too simplistic view of the complexity of “host-microbe-microbe” interactions.

## Author contributions

EP was a guest associate editor of the Research Topic and wrote the paper text. J-CS and JN were guest associate editors of the Research Topic and edited the text. All authors contributed to the article and approved the submitted version.

## Funding

EP is supported by the National Institutes of Health (NIGMS 1T34GM145503). JS is supported by Spanish Ministry of Science and Innovation (AEI/10.13039/501100011033 under grant PID2019-105117RR-C2). JN is supported by the Genesis Biotechnology Group.

## Conflict of interest

The authors declare that the research was conducted in the absence of any commercial or financial relationships that could be construed as a potential conflict of interest.

## Publisher’s note

All claims expressed in this article are solely those of the authors and do not necessarily represent those of their affiliated organizations, or those of the publisher, the editors and the reviewers. Any product that may be evaluated in this article, or claim that may be made by its manufacturer, is not guaranteed or endorsed by the publisher.
